# Recipient and donor thrombophilia and the risk of portal venous thrombosis and hepatic artery thrombosis in liver recipients

**DOI:** 10.1186/1471-230X-11-130

**Published:** 2011-11-28

**Authors:** Rosa Ayala, Joaquín Martínez-López, Teresa Cedena, Rosalía Bustelos, Carlos Jimenez, Enrique Moreno, Carmen Ribera

**Affiliations:** 1 Hematology Department, 12 De Octubre University Hospital, Madrid, Spain; 2 General Surgery Alimentary Tract and Abdominal Organ Transplantation Department, 12 De Octubre University Hospital, Madrid, Spain; 3 Hematology Department, Sureste Hospital (Arganda), Madrid, Spain; 4 Complutense De Madrid University, Madrid, Spain

## Abstract

**Background:**

Vascular complications, such as HAT, are an important cause of graft loss and recipient mortality. We aimed to characterize post-transplant thrombotic events in a cohort of liver transplant recipients, and identify independent risk factors for these complications.

**Methods:**

We conducted a thrombophilic study of 293 orthotopic liver transplants performed in the Digestive Surgery Department of the 12 de Octubre Hospital (Madrid, Spain) between January 2001 and December 2006.

**Results:**

The most frequent post-transplant thrombotic events were HAT (9%) and PVT (1.7%). The one variable associated with post-transplant thrombotic event was a high fibrinogen level in the global cohort of liver transplantation. But toxicity as event post-OLT has been associated with post-transplant thrombotic event in the retrospective group and high fibrinogen level and low protein C levels were associated post-transplant thrombotic event in the prospective group. Liver disease relapse (HR 6.609, p < 0.001), high levels of FVIII (HR 1.008, p = 0.019)) and low levels of antithrombin (HR 0.946, p < 0.001) were associated with poor overall survival (OS).

In conclusion, high fibrinogen and decreased protein C levels were associated with allograft thrombosis. Further studies are required in order to assess the clinical relevance of these parameters in prospective studies and to study the effect of anticoagulation prophylaxis in this group of risk.

## Background

Vascular complications after orthotopic liver transplantation (OLT) are one of the most feared problems that frequently result in graft and patient loss. Early hepatic artery thrombosis (eHAT) is a major complication following liver transplantation and since Starzl's first report on human liver transplantation it is recognized as an important cause of graft loss and mortality [1,2].

HAT is generally believed to result primarily from surgical techniques (kinking, stenotic anastomosis). However, some studies show that eHAT is also associated with no-surgical factors, such as donor age [3], rejection, and sluggish flow through the hepatic artery [1,4,5]. The presence of Factor V Leiden in the graft [6,7], or changes in the hemostatic system [8-11], are also believed to play a role.

Recently a meta-analysis of the incidence, risk factors and outcome of eHAT after liver transplantation showed the risk factors of eHAT to be cytomegalovirus (CMV) mis-match, re-transplantation, arterial conduits, prolonged operation time, low recipient weight, variant arterial anatomy, and low volume transplant centers [12]. However, the determinants of thrombophilia were not included in this study.

We aimed to characterize post-transplant thrombotic events in a cohort of liver transplant recipients, and to identify possible parameters that could lead to HAT and could be responsible for graft loss due to thrombosis in extended thrombophilia study.

## Methods

### Patient population

Between January 2001 and December 2006, 441 orthotopic liver transplants (OLT) were performed in the Digestive Surgery Department of the 12 de Octubre Hospital (Madrid, Spain). There were 441 grafts and 437 liver recipients. Before initiating this study, 74 recipients had died (14 of whom demonstrated a thrombo-embolic event [TE], and in two of these this was considered to be the cause of death). In another 74 cases, we were unable to obtain laboratory results because the referred patients did not attend follow-up visits at our study center, or did not sign the consent form. Wherever possible all patients are lab tested for hypercoagulation study irrespective of any presence of thrombophilia.

We have therefore reported analytical thrombophilia results for 293 patients. As this study was initiated in July 2004, the majority of patients had already undergone OLT. Thus, blood samples for these patients could only be taken at their post-transplant follow-up visits. The present work is made up of a retrospective study that includes 167 patients up to July 2004 and a later prospective study that includes another 126 patients. Blood samples were taken at a median of 26.3 months and 3.1 months post-transplantation in the retrospective and prospective studies, respectively. Patients of the retrospective group were evaluated by medical reports. From 2004, the evaluation was prospective for all patients, monthly of quarterly. Clinical and analytical studies were utilized for evaluation of patients. Abdominal Doppler, computed tomography scan, or hepatic biopsies were carried out whenever necessary. The analysis has been done separately in both the prospective and retrospective groups. The study was approved by our Ethical Institutional Review Board and all patients gave their consent for blood samples to be further processed. Data on thrombophilia risk factors were collected by personal interview, or by reviewing the medical history.

### Recipients

The study population comprised 293 males and 144 females ranging from 1 month to 74.5 years old (median 51.73 y). Two-hundred and eighty-six cases were primary OLTs, 46 were secondary OLTs, and 9 cases were tertiary OLTs. The cause of liver transplantation was: 20 HBV (hepatitis B virus), 57 HCV (hepatitis C virus), 40 HBV and HCV, 53 Ethanol, 8 Autoimmune, 7 Toxicity, 68 Tumor, 16 Alpha 1 antitrypsin deficits, 14 Biliary atresia. We use "toxicity" to mean liver damage probably caused by immunosuppressant therapy diagnosed by liver biopsy. Three patients have received anticoagulation therapy pre-OLT and 11 patients anticoagulation therapy post-OLT. These patients were considered separately. Post-OLT immunosuppressant regimen was triple therapy: FQ (tacrolimus) or CsA (cyclosporine), MMF (mycophenolate mofetil) and Cc (corticosteroids). Corticosteroid is administred for 1 month post-OLT (1mg/kg/day), and is later reduced to zero.

### Donors

Forty-one live donor liver transplantation (LDLT), and 252 deceased donor liver transplantation (DDLT) donors were evaluated. Eighty-six partial graft livers were employed in the cases of DDLT. LDLT donors comprised 24 males and 17 females with a median age of 32 y (range, 19-52 y). LDLT donors were assessed before OLT using functional coagulation and genetic tests. In DDLT donors, only genetic tests were carried out. Genetic studies were performed in 293 recipients and 222 donors (181 deceased, and 41 living donors). Seventy one donor samples were not suitable.

## Methods

### Genetic thrombophilia study

The extracted genomic DNA was processed to detect Factor V Leiden, prothrombin G20210A and methylenetetrahydrofolate (MTHFR) C667T mutations, using real-time PCR (polymerase chain reaction) with hybridization probes in a light-cycler (Roche Diagnostics, Mannheim, Germany). We have tested these genetic mutations in a subgroup of donors and recipients appropriately matched and the tests were performed in the donors on blood samples collected at the time of procurement.

### Functional coagulation and hemostatic tests

The following panel of tests was run: prothrombin activity (PA) (Dade Behring, Marburg, Germany) (This test is the prothrombin time expressed as a percent and for their interpretation, the normal range is 75-130% and a low percent indicates a prolonged prothrombin time), activated partial thromboplastin time (APTT) (Biomérieux, Craponne, France), and thrombin clotting time (TT) (Biomérieux, Craponne, France). Fibrinogen concentration was measured with a coagulometric assay using the Clauss method (Biomérieux, Craponne, France). AT (Chromogenix, Mölndal, Sweden), PC (Roche Diagnostics, Mannheim, Germany), and PS (Roche Diagnostics, Mannheim, Germany) activities were measured using chromogenic substrate assays. PS antigen levels were assessed by enzyme linked immunosorbent assay (ELISA) (Diagnostic Grifols, Barcelona, Spain). Levels of homocysteine were measured by fluorescent polarization immunoassay (FPIA) using the Abbott Laboratories IMX System (Abbott park, Illinois, USA). Hyperhomocysteinemia was defined as a homocysteine level > 14 μmol/L. Activated PC resistance (APCR) was measured by Coatest APC resistance (Chromogenix, Mölndal, Sweden), and IgG and IgM anti-cardiolipin antibodies were measured using the *APhL*^® ^ELISA Kit (Grifols Engineering, Barcelona, Spain). Platelet counts were done on the ADVIA 2120 (Siemens AG, Munich, Germany). Factor VIII was measured by one-stage clotting assays (Dade Behring, Marburg, Germany) and was said to be increased at levels above 150 IU/dL.

### Follow-up

Data on the clinical evolution of recipients were collected by searching their medical records. Deep vein thrombosis (DVT), organ vein thrombosis (PVT, HAT, renal vein thrombosis, mesenteric thrombosis), pulmonary embolism (PE), myocardial infarction, ischemic stroke, and peripheral arterial disease, were defined as a pre-transplant thrombosis when the event occurred before liver transplantation, and were defined as post-transplant thrombosis when the event occurred after liver transplantation. Thrombotic events were diagnosed by Doppler ultrasonography, computed tomography scan, or hepatic biopsies. The follow-up period for donors and recipients began on the date of liver transplantation, and ended on the date of death, date of re-transplant, or the end of study (March 1, 2007). Follow-up periods ranged from 15 d to 8 y (median: 27 months).

### Statistical analyses

The Pearson's chi-square (χ2) statistic (or Fisher's exact test) and the Student's t test (or Kruskal-Wallis test) were used to test for differences in the distribution of dichotomous variables, and for differences in the mean values of continuous distributions. Forward stepwise logistic regression was used to identify independent risk factors for PVT and HAT. The following variables collected at diagnosis were included in the database: gender (male/female), age (both as a continuous variable, and grouping patients over and under 16 y of age), the existence of clinical thrombophilia risk factors, the original liver disease, and post-transplantation events.

Overall survival (OS) was calculated from the day of the liver transplantation to death. Kaplan-Meier life tables were constructed for survival data and were compared by means of the log-rank test. A census of the surviving patients was taken on March 1, 2007. The median follow-up time was 21.7 months (range 1-184.6 months). Results with a *P *value less than 0.05 were considered significant.

## Results

### Thrombophilia in the study population

In recipients, the prevalence of the heterozygote Factor V Leiden mutation was 7 of 293 (2.4%), the heterozygote G20210A prothrombin mutation was 13 (4.5%), and the homozygote C677T MTHFR mutation was 41 (14.1%). In donors, the prevalence of the heterozygote Factor V Leiden mutation was 4 out of 222 (1.6%), the heterozygote G20210A prothrombin mutation was 7 (2.8%), and the homozygote C677T MTHFR mutation was 33 (13%). 

Pathology results in this population showed the following: 32 cases (10.9%) had PA < 80%; 34 (11.6%) had a prolonged APTT (> 36”) and 7 (2.4%) had a reduced APTT (< 26”); 19 cases (6.5%) had fibrinogen > 400 mg/dL, and 6 (2%) had fibrinogen < 150 mg/dL; 21 cases (7.2%) had AT < 80%; 143 (48.8%) had FVIII > 150%, and one (0.34%) had FVIII < 65%; 30 (10.2%) had PC < 65%; 53 (18.1%) had PS < 65%; 70 (23.9%) had plasma homocysteine >14 µmol/L. Anti-cardiolipin IgG or IgM was high in 25 cases (8.5%) (9 IgG, and 17 IgM), and 4 (1.4%) were APC-resistant after OLT (≤ 2). 

7 factor V Leiden carrier recipients received a no-Leiden liver and all factor V Leiden carrier recipients were not APC-resistant after transplantation (APCR after OLT: 2.91, 3.56, 2.36, 3.8, 2.32, 3.17, and 3.23) as expected. Nevertheless neither of the 2 recipients analyzed with a factor V Leiden graft were APC-resistant after the transplant (2.91 and 2.24). Three of four no-Leiden carrier recipients who were APC-resistant after OLT showed higher FVIII levels (345%, 219%, 174%), but only one donor could be tested, and that donor was found to have a wild-type FV genotype. The Coatest assay used not include factor V deficient plasma, then the APC-resistance can be caused by high factor VIII. 

### Thrombotic events in the study population

**Recipients.- **Fifty-eight (12.8%) patients developed a post-transplant thrombotic event (1 ischemic stroke, 6 DVT-PE, 9 PVT, 41 HAT, and 1 peripheral arterial disease).

**Living donors.- **After surgery, donors' liver function recovered well and no bleeding events were recorded. Donors did not experience thrombotic events, neither during the immediate post-transplant nor during the follow-up periods [13].

### Thrombotic events after orthotopic liver transplantation and clinical thrombophilia risk factors

The most frequent post-transplant thrombotic event was HAT, with a frequency of 9% (41/441 cases, 63.5% of all post-transplant thrombotic event), and the second most frequent was PVT (8/441 cases), and one with HAT and PVT. Of all 50 graft thrombosis cases, 38 were secondary liver transplantations. HAT and PVT were most frequent when the recipient was a child (37 of the 58 liver recipients with post-transplant thrombotic event were children, versus 42 of 379 without post-transplant thrombotic event, *P *< 0.001). The patients with biliary atresia, all children, showed a higher frequency of post-transplant thrombotic event (15 cases of 58 with post-transplant thrombotic event, versus 18 of 379 without post-transplant thrombotic event, *P *< 0.001). The presence of toxicity was associated with post-transplant thrombotic event (19 cases of 56 with post-transplant thrombotic event versus 78 of 368 without post-transplant thrombotic event, p = 0.035) but not the presence of tumours. (Additional File 1, Table S1).

In the 6 cases with venous thromboembolic processes (other than PVT or HAT) in the post-transplant period, the thrombophilic factors were: case 1, complicated post-surgery period (metabolic diseases, infections, critical illness neuropathy); case 2, smoking and previous surgery; case 3, diabetes and tumour associated; cases 4 and 5, relapse HBV or HCV and tumour associated; case 6, with previous pregnancy complications, she developed acute lymphoblastic leukemia and a DVT with slightly low antithrombin. As expected, an increased frequency of subjects with associated cancer was found among patients with no-liver related post-transplant thrombotic event.

### Correlation between thrombotic events and the thrombophilia study

The laboratory findings of the two groups with or without post-transplant thrombotic event are reported in Table 1. Only higher platelet counts (*P *< 0.001) and fibrinogen levels (*P *< 0.001) were associated with post-transplant thrombotic event in univariate analysis.

**Table 1:** Hypercoagulability study in association with post-transplant thromboses

	With thrombosis post-transplant (26 Cases)	Without thrombosis post-transplant (267 Cases)	Significance
**RECIPIENT STUDY**			

**Platelets **(×10^9^/L)	327.27 ± 88.0	264.89 ± 73.7	**p < 0.001**

**PA **(%)	101.96 ± 21.5	101.79 ± 16.4	p = 0.962

**APTT **(seconds)	31.24 ± 6.1	31.08 ± 4.4	p = 0.866

**TT **(seconds)	13.8 ± 2.2	14.22 ± 1.9	p = 0.301

**Fibrinogen **(mg/dL)	327.3 ± 88.7	264.89 ± 73.7	**p < 0.001**

**AT **(U/dL)	107.57 ± 18.3	99.57 ± 15.3	**p = 0.013**

**PC chromogenic **(U/dL)	61.34 ± 41.9	74.69 ± 39.8	p = 0.069

**PC anticoagulant **(U/dL)	94.68 ± 18.99	102.61 ± 27.5	p = 0.065

**PS free antigen **(U/dL)	84.0 ± 12.7	77.85 ± 14.2	**p = 0.038**

**PS functional **(U/dL)	95.4 ± 24.9	83.44 ± 22.3	**p = 0.011**

**PS total **(U/dL)	90.0 ± 9.0	93.9 ± 18.3	p = 0.716

**Factor VIII **(UI/dL	168.77 ± 60.8	153.95 ± 56.9	p = 0.209

**ACA IgG **(uFl)	5.84 ± 5.0	7.56 ± 16.49	p = 0.605

**ACA IgM **(uFl)	11.24 ± 18.64	9.3 ± 14.68	p = 0.539

**Plasma homocysteine **(μmoles/L)	13.79 ± 3.35	14.31 ± 8.7	p = 0.585

**Factor V Leiden mutation **(N°) **(heterozygous) in recipient**	1/26	6/264	p = 0.618

**Prothrombin 20210A mutation **(N°) **(heterozygous) in recipient**	0/26	13/264	p = 0.247

**C677T MTHFR mutation (N°) (wild type/heterozygous/homozygous) in recipient**	13/8/4	102/117/37	p = 0.402

**APCR**	2.86 ± 0.51	2.92 ± 0.51	p = 0.582

**DONOR STUDY**			

**Factor V Leiden mutation **(N°) **(heterozygous) in donor**	0/26	4/196	p = 0.454

**Prothrombin 20210A mutation **(N°) **(heterozygous) in donor**	1/26	6/194	p = 0.865

**C677T MTHFR mutation (N°) (wild type/heterozygous/homozygous) in donor**	9/14/3	63/101/28	p = 0.915

PA (pro-thrombin activity); APTT (activated partial thromboplastin time); TT (thrombin clotting time); AT (anti-thrombin); PC (protein C); PS (protein S); ACA (anticardiolipin antibody); APCR (activated protein C resistance); (MTHFR) 5,10-methylenetetrahydrofolate reductase.

Live donor candidates were excluded from donation if they presented any abnormalities in the hemostatic study, due to genetic mutations, anticoagulant protein deficiencies, factor VIII deficiency or high levels of anticardiolipin antibodies [13].

Lower recipient platelets count and fibrinogen, AT, PC, PS were associated with liver disease relapse but not the FVIII level. (see Additional File 2, Table S2).

### Early HAT versus Late HAT

Of the 41 post-transplant HAT, 22 events occurred in the early post-operatory period, and the remaining 19 took place after at least 3 months post-surgery. Frequent characteristics of the early HAT group were: pediatric recipients younger than 2 years old (13 cases), hemodynamic problems during surgery (4 cases), infections (4 cases), haemolytic anemia (2 cases), previous venoocclusive disease (3 cases), and no-heart-beating donor (2 cases). Frequent characteristics of the late HAT group were cirrhosis due to HCV reactivation (10 cases), graft rejection (6 cases), severe infections (3 cases), and hepatocellular tumour (6 cases).

Considering only the population with post-transplant thrombosis, partial graft resection was associated with early HAT (13 of 22 early HAT were partial graft resection versus 2 of 19 late HAT, p = 0.024). Certain post-transplant events were associated with late HAT, such as tumor (6 of 19 late HAT were tumor associated versus 2 of 22 early HAT, p = 0.018), cirrhosis recurrence (10 of 19 late HAT were associated with recurrence versus 4 of 22 early HAT, p = 0.002), and also low PC (mean PC was 77% and 90% with and without recurrence, respectively, *p *= 0.006). Other events such as toxicity, showed a tendency to be associated with late HAT (p = 0.064). Other clinical and laboratory variables were not especially associated with early or late HAT.

### Multivariate study

In the multivariate study the one variable associated with post-transplant thrombotic event was a high fibrinogen level (continuous variable) (HR 1.008; *P = *0.01), in the global cohort of liver transplantation (Table 2). But toxicity as event post-OLT has been associated with post-transplant thrombotic event in the retrospective group (Table 3) and high plasma levels of fibrinogen recipient and low plasma levels of protein C were associated post-transplant thrombotic event in the prospective group (Table 4).

**Table 2:** Logistic Regression analysis of factors associated with post-transplant thrombosis IN GLOBAL COHORT OF LIVER TRANSPLANTATION.

Variable	p	Hazard Ratio (CI)
CHILD/ADULT RECIPIENT	0.138	
TOXICITY AS EVENT POSTRANSPLANT (YES/NO)	0.106	
ASSOCIATED TUMOR	0.952	
CAUSE OF LIVER TRANSPLANTATION	0.443	
FIBRINOGEN RECIPIENT (CONTINUOUS VARIABLE)	**< 0.001**	**1.010 (1.004-1.016)**
PLATELET COUNT RECIPIENT (CONTINUOUS VARIABLE)	0.443	
FVIII LEVEL RECIPIENT (CONTINUOUS VARIABLE)	0.629	
ATIII RECIPIENT (CONTINUOUS VARIABLE)	0.237	
FREE PROTEIN S RECIPIENT	0.473	
PROTEIN C RECIPIENT	0.052	

The one variable associated with post-transplant TE was a high fibrinogen level (continuous variable) (HR 1.008; *P = *0.01), in the global cohort of liver transplantation.

**Table 3:** Logistic Regression analysis of factors associated with post-transplant thrombosis IN RETROSPECTIVE COHORT OF LIVER TRANSPLANTATION.

Variable	p	Hazard Ratio (CI)
CHILD/ADULT RECIPIENT	0.213	
TOXICITY AS EVENT POSTRANSPLANT (YES/NO)	**0.019**	**4.484 (1.284-15.664)**
ASSOCIATED TUMOR	0.544	
CAUSE OF LIVER TRANSPLANTATION	0.985	
FIBRINOGEN RECIPIENT (CONTINUOUS VARIABLE)	0.115	
PLATELET COUNT RECIPIENT (CONTINUOUS VARIABLE)	0.416	
FVIII LEVEL RECIPIENT (CONTINUOUS VARIABLE)	0.646	
ATIII RECIPIENT (CONTINUOUS VARIABLE)	0.087	
FREE PROTEIN S RECIPIENT	0.635	
PROTEIN C RECIPIENT	0.261	

Only toxicity as event post-OLT has been associated with post-transplant TE in the retrospective group

**Table 4:** Logistic Regression analysis of factors associated with post-transplant thrombosis IN PROSPECTIVE COHORT OF LIVER TRANSPLANTATION.

Variable	p	Hazard Ratio (CI)
CHILD/ADULT RECIPIENT	**0.075**	**0.056 (0.002-279)**
TOXICITY AS EVENT POSTRANSPLANT (YES/NO)	0.096	
ASSOCIATED TUMOR	0.053	
CAUSE OF LIVER TRANSPLANTATION	0.253	
FIBRINOGEN RECIPIENT (CONTINUOUS VARIABLE)	**0.023**	**1.023 (1.003-1.044)**
PLATELET COUNT RECIPIENT (CONTINUOUS VARIABLE)	0.743	
FVIII LEVEL RECIPIENT (CONTINUOUS VARIABLE)	0.918	
ATIII RECIPIENT (CONTINUOUS VARIABLE)	0.063	
FREE PROTEIN S RECIPIENT	0.941	
PROTEIN C RECIPIENT	**0.025**	**0.950 (0.909-0.994)**

High plasma levels of fibrinogen recipient and low plasma levels of protein C were associated post-transplant TE in the prospective group.

### Survival analysis

**Figure 1:**
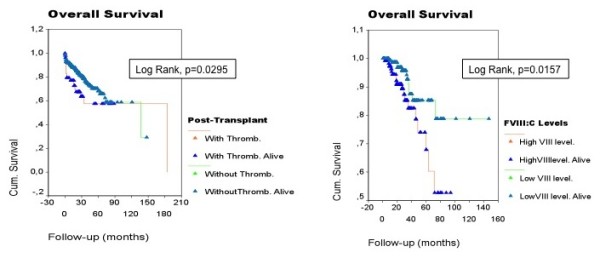
**Liver transplant recipient overall survival (OS)**. A. Post-transplant thrombosis was associated with a poor outcome (*P *< 0.03). B. High FVIII:C levels were associated with a poor outcome: the median OS was 69.6 and 132.1 months in groups with high and low FVIII:C, respectively (*P *< 0.003). Median follow-up was 21.7 months (range 1-184.6 months).

**Figure 2:**
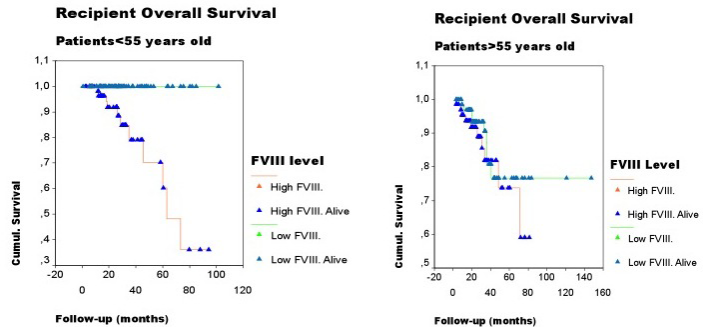
**High FVIII:C levels and Recipient Overall Survival**. High FVIII:C levels were associated with a poor outcome separately in both young and elderly groups (Log Rank 8.56; p = 0.0034).

To determine whether the presence of post-transplant thrombotic event influenced recipient survival, we performed a survival analysis (Figure 1). The results indicated that only post-transplant thrombotic event was associated with a poor outcome (median OS was 136.5 and 184.6 months in groups with post-transplant thrombotic event and without post-transplant thrombotic event, respectively, *P *< 0.03). Figure 2 shows the influence of level FVIII on the overall survival separately in both young and elderly groups. Liver disease relapse (no/yes) (HR 6.609; 95%CI, 2.674-16.335), high levels of FVIII (continuous variable) (HR 1.0008; 95%CI, 1.001-1.015) and low levels of antithrombin (continuous variable) (HR 0.946; 95%CI, 0.921-0.971) have been independently associated with mortality from the time of liver transplantation in a multivariable Cox regression model (Table 5).

**Table 5:** Multiple Cox Regression Analysis for OS in a cohort of liver transplantation.

Variable	p	Hazard Ratio (CI)
AGE (CONTINUOUS VARIABLE)	0.059	
PARTIAL GRAFT (YES/NO)	0.919	
DONOR TYPE (LIVE OR DECEASED)	0.500	
CAUSE OF LIVER TRANSPLANTATION	0.719	
TOXICITY AS EVENT POST-TRANSPLANT	0.479	
**LIVER DISEASE RELAPSE AS EVENT POST-TRANSPLANT**	**< 0.001**	**6.609 (2.674-16.335)**
THROMBOSIS AS EVENT POST-TRANSPLANT	0.600	
TUMOR AS EVENT POST-TRANSPLANT	0.303	
**FVIII LEVEL RECIPIENT(CONTINUOUS VARIABLE)**	**0.019**	**1.008 (1.001-1.015)**
**ANTITHROMBIN LEVEL RECIPIENT (CONTINUOUS VARIABLE)**	**< 0.001**	**0.946 (0.921-0.971)**
PROTEIN C RECIPIENT	0.931	
PROTEIN S RECIPIENT	0. 388	
FIBRINOGEN LEVEL RECIPIENT	0.264	
PLATELET COUNT RECIPIENT	0.233	

Relapse of liver disease (HR 6.609), high levels of FVIII (HR 1.0008) and low levels of antithrombin (HR 0.946) have been independently associated with mortality from the time of liver transplantation in a multivariable Cox regression model. Split Liver Transplant: In some cases, it is possible to divide the donor liver into two parts and transplant the parts into two recipients or in the case of the live donor liver transplant too.

## Discussion

Post-transplant HAT and/or PVT increase the potential for graft loss. The frequency of post-transplant HAT has been previously recorded as 4.5% to 6%[14,15]. In our study, the most frequent post-transplant thrombotic event was HAT, with a frequency of 9% (41/441 cases, 63.5% of all post-transplant thrombotic event). However, there is no extensive thrombophilia study in a large cohort of liver recipients. We evaluated the thrombophilia-associated risk factors and their clinical impact was assessed. The partial retrospective design and heterogeneity of the study population are limitations of our study.

Liver transplantation itself also changes the dynamics of coagulation, with a pro-coagulant pattern in the first weeks post-transplant. Due to the thrombophilia study was carried out between 1.2 months to 4.1 months post-transplant, thrombophilic abnormalities in immediate post-surgery, with influence in the development of early HAT, could be underestimated. Nevertheless, we detected thrombophilic abnormalities that were still evident during the first 3 months post-surgery or during the late post-transplant period, and which were associated with HAT.

The importance of genetic thrombophilia risk factors in PVT has been investigated in several studies [16-19] but all of them have excluded cases with local risk factors. The incidence of Factor V Leiden, prothrombin mutation, decreased PC, decreased PS, and decreased AT has been recorded between 3-30%, 3-22%, 0-26%, 2-43%, and 1-26%, respectively. In our study the incidence of decreased PC, decreased PS, and decreased AT was 2.4%, 4.5%, 10.2%, 18%, and 7.2%, respectively. The incidences of decreased PC, PS and AT were higher than that observed in the general population. Nevertheless, these deficiencies coincided with deteriorating liver function in most cases.

Most of the coagulation changes due to genetic mutations in the recipient are corrected by liver transplantation. Of the 293 patients screened for genetic analysis of Factor V Leiden and who underwent functional studies of APCR after OLT, 7 heterozygous carriers of Factor V Leiden displayed a loss of APCR after transplantation, as previously described [20-22]. In our study, recipients of graft Factor V Leiden did not acquire APCR, probably because the platelet derived Factor V originates from the plasma pool [23-25]. However, these results do not correlate with previous work which states that APCR associated with a Factor V Leiden donor is fully transferable to the graft recipient [22].

Some of the variables associated with post-transplant thrombotic event could relate to difficulties in surgical technique, such as in a child recipient, and also their primary cause of liver transplantation, biliary atresia. In a recent study [26], post-transplant complication rates, including the re-operation rate, were higher in the younger group. In our study, fibrinogen level was the one variable independently associated with post-transplant thrombotic event in the global cohort of liver recipients. Previously this factor has been associated both with thrombosis and inflammation. A meta-analysis showed that risk of coronary heart disease may increase 1.8 fold for 1 g/L of increased fibrinogen, independent of traditional risk factors [27]. But, in the retrospective cohort of recipients, the toxicity was the only variable independently associated. This factor could be involved in graft thrombosis due to its role in endothelial injury and inflammation. Plasma levels of fibrinogen (high) and protein C recipient (low) were associated post-transplant thrombotic event in the prospective group. Low plasma levels of protein C recipient was associated, in this work, with liver disease relapse (Additional File 2, Table S2), and this fact could be a consequence of disbalance of coagulation factors in terminal cirrhosis [28]. A previous paper had shown that PC was decreased at day 7 (p = 0.04) and day 30 (p = 0.009) in DDLT and DDRT/LRT groups with complications, respectively [29]. Nevertheless, high plasma fibrinogen levels are not a consequence of liver function alteration, and could be an important pro-thrombotic factor in development graft thrombosis that has not been previously described.

Our results were in agreement with previous studies [12] showing that the presence of post-transplant thrombotic event, HAT in the majority of cases, in this liver recipient cohort influenced recipient overall survival. High factor VIII was associated with a poor outcome, as previously reported. Levels of factor VIII, a potent pro-coagulant involved in thrombin generation, increased progressively with Child-Pugh Score (from Child-Pugh class A to C)[30]. A correlation between the severity of liver disease and von Willebrand factor (VWF) plasma antigen levels has been previously documented [31,32]. In agreement with other authors [33], we emphasize the need for studies to evaluate the clinical relevance of including thrombotic risk factors (such as factor VIII associated to poor outcome) in the Prognostic scores ej. Model for End-stage Liver Disease (MELD).

## Conclusion

To the best of our knowledge, this is the first extensive thrombophilia study in a large cohort of liver recipients. Therefore, our study does not support universal long thrombophilia screening in liver recipients. High fibrinogen and decreased protein C are associated with allograft thrombosis. The practical impact these findings could be the need to include parameters such as fibrinogen and protein C levels as risk factors of graft thrombosis and to assess the effect of anticoagulation prophylaxis in this group of risk. Further studies are required in order to assess the clinical relevance of these parameters in prospective studies.

## Competing interests

The authors declare that they have no competing interests.

## Authors' contributions

Contribution: RA, JML and CR designed research; RA and RB performed molecular and functional experiments; RA, JML, TC, RB, CJ, EM, and CR analyzed and interpreted data; RA designed, performed, and wrote the manuscript; JML and CR supervised the research and critically revised the manuscript. All authors read and approved the final manuscript.

## Pre-publication history

The pre-publication history for this paper can be accessed here:

http://www.biomedcentral.com/1471-230X/11/130/prepub

## Supplementary Material

Additional file 1 **Table S1. Clinical thrombophilic risk factors in association with post-transplant thromboses**. Click here for file

Additional file 2 **Table S2. Mean Comparison (T Student Test) of study variables in function of recidive as event post-transplant**. Click here for file
