# Supplementary material for: Recipient and donor thrombophilia and the risk of portal venous thrombosis and hepatic artery thrombosis in liver recipients
Source: BMC Gastroenterol. 2011 Nov 28;11:130. doi: 10.1186/1471-230X-11-130 (PMC3287260; doi:10.1186/1471-230X-11-130)
Supplement: Additional file 1 — Table S1. Clinical thrombophilic risk factors in association with post-transplant thromboses. [file 1471-230X-11-130-S1.DOC]

|  |  | | |  |  |  |  | |  | |  |  |
| --- | --- | --- | --- | --- | --- | --- | --- | --- | --- | --- | --- | --- |
|  | **Table. S1** | | | **Clinical thrombophilic risk factors in association with post-transplant thromboses.** | | | | | | | | |
|  |  | | | **With post-transplant thrombosis** | | | | **Without post-transplant thrombosis** | | **Significance** | | |
|  |  | | |  | | | |  | |  | | |
| Child/ adult recipient | | | | 37/19 | | | | 42/335 | | **p<0.001** | | |
| Male/ female recipient | | | | 32/24 | | | | 262/120 | | p=0.089 | | |
| Associated tumor (Yes/No) | | | | 11/28 | | | | 75/160 | | p=0.644 | | |
| Diabetes mellitus and/or high lipid (Yes/No) | | | | 14/26 | | | | 93/153 | | p=0.734 | | |
| Inflammation disease (Yes/No) | | | | 1/33 | | | | 0/213 | | p=0.012 | | |
| Inmobility (Yes/No) | | |  | 2/32 | | | | 12/198 | | p=0.969 | | |
| Surgical intervention (Yes/No) | | | | 22/21 | | | | 132/119 | | p=0.863 | | |
| Obesity (Yes/No) | | |  | 3/33 | | | | 8/204 | | p=0.219 | | |
| Nephrotic syndrome (Yes/No) | | | | 0/34 | | | | 14/199 | | p=0.124 | | |
| Myeloproliferative syndrome (Yes/No) | | | | 0/35 | | | | 2/214 | | p=0.568 | | |
| Trauma (Yes/No) | | |  | 2/34 | | | | 14/201 | | p=0.828 | | |
| Deceased donor (Yes/No) | | |  | 7/49 | | | | 35/342 | | p=0.448 | | |
| Partial graft resection | | |  | 15/41 | | | | 72/305 | | p=0.180 | | |
| Cause of Liver Transplantation | | | |  | | | |  | | **p<0.0001** | | |
|  | BHV | |  | 3 | | | | 18 | |  | | |
|  | CHV | |  | 20 | | | | 188 | |  | | |
|  | Ethanol | |  | 13 | | | | 78 | |  | | |
|  | Autoimmune | |  |  | | | | 11 | |  | | |
|  | Toxicity | |  |  | | | | 8 | |  | | |
|  | Tumor | |  |  | | | | 14 | |  | | |
|  | Cryptogenetic | | | 1 | | | | 15 | |  | | |
|  | Vascular | |  |  | | | | 1 | |  | | |
|  | Infrequent (Alpha-1-antitrypsin deficiency) | | | 4 | | | | 24 | |  | | |
|  | Biliary atresia | | | 15 | | | | 18 | |  | | |
| Events post-transplantation | |  | |  | | | |  | |  | | |
|  | Re-infection (Yes/No) |  | | 11/41 | | | | 72/264 | | p=0.964 | | |
|  | Toxicity (Yes/No) |  | | 19/37 | | | | 78/290 | | **p=0.035** | | |
|  | Tumor (Yes/No) |  | | 8/48 | | | | 27/342 | | p=0.077 | | |
|  | Rejection (Yes/No) |  | | 20/36 | | | | 132/240 | | p=0.973 | | |
|  | Re-transplant (Yes/No) | | | 24/30 | | | | 19/322 | | **P<0.001** | | |
|  | Recurrence (Yes/No) |  | | 14/42 | | | | 140/231 | | p=0.069 | | |
