# Supplementary material for: Recipient and donor thrombophilia and the risk of portal venous thrombosis and hepatic artery thrombosis in liver recipients
Source: BMC Gastroenterol. 2011 Nov 28;11:130. doi: 10.1186/1471-230X-11-130 (PMC3287260; doi:10.1186/1471-230X-11-130)
Supplement: Additional file 2 — Table S2. Mean Comparison (T Student Test) of study variables in function of recidive as event post-transplant. [file 1471-230X-11-130-S2.DOC]

| Variable | Recidive Liver Disease | N | Media | SD | Significance |
| --- | --- | --- | --- | --- | --- |
| Platelets Count Recipient (x10^9(^/L) | Non | 170 | 179852,9412 | 84030,33136 | <0.001 |
|  | Yes | 119 | 137215,1261 | 65490,37015 |  |
| PA (%) | Non | 175 | 102,8457 | 16,73884 | NS |
|  | Yes | 117 | 100,0000 | 16,93751 |  |
| APTT (seconds) | Non | 175 | 30,6000 | 4,35230 | 0.025 |
|  | Yes | 117 | 31,8205 | 4,78614 |  |
| Fibrinogen (mg/dl) | Non | 176 | 279,8239 | 79,85442 | 0.006 |
|  | Yes | 118 | 254,8559 | 69,08749 |  |
| Plasma Homocystein (µml/L) | Non | 151 | 15,1914 | 10,35173 | 0.03 |
|  | Yes | 112 | 13,1196 | 4,59061 |  |
| AT (U/dl) | Non | 176 | 102,2443 | 13,79575 | 0.011 |
|  | Yes | 118 | 97,5000 | 17,85973 |  |
| Factor VIII (UI/dl) | Non | 176 | 154,2898 | 56,60652 | NS |
|  | Yes | 118 | 155,3220 | 57,35731 |  |
| PC anticoagulant (U/dl) | Non | 175 | 108,3371 | 24,54261 | <0.001 |
|  | Yes | 117 | 92,6325 | 27,83715 |  |
| PS free antigen (U/dl) | Non | 174 | 81,1437 | 14,37029 | <0.001 |
|  | Yes | 117 | 74,0598 | 12,78623 |  |

**TABLE S2. Mean Comparison (T Student Test) of study variables in function of recidive as event post-transplant.**
